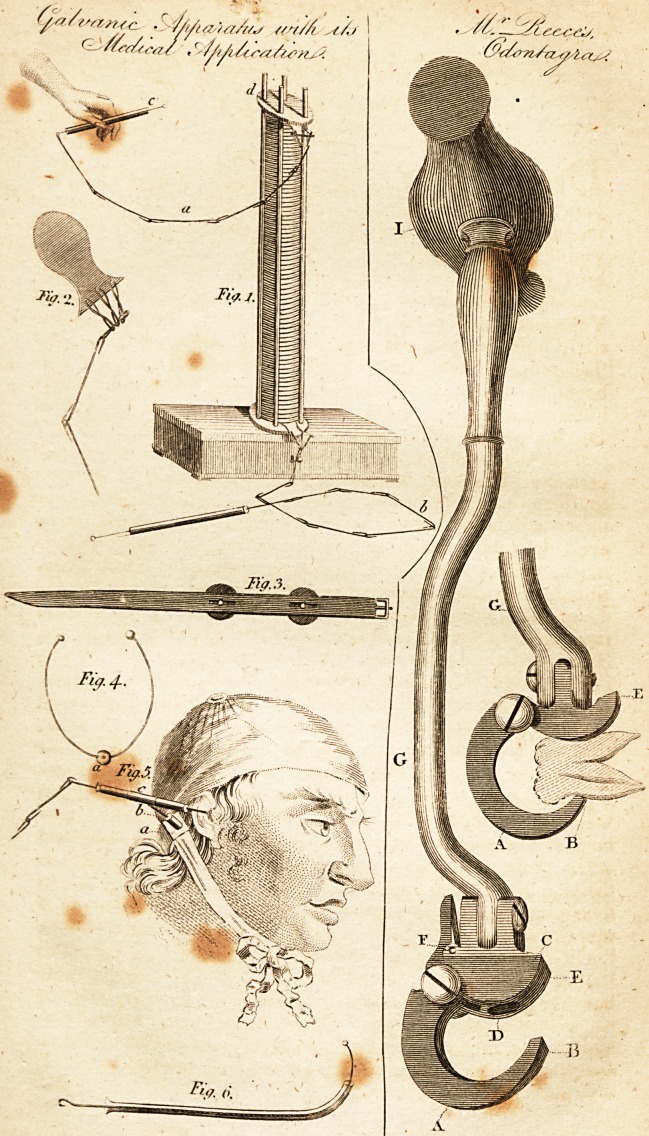# Dr. Augustin, on the Medical Use of Galvanism

**Published:** 1802-03

**Authors:** 


### *Dr. Augustin, on the Medical Use of Galvanism.*

[ With an Engraving. ]

DR. AUGUSTIN, of Berlin, author of feveral ufeful and judicious compilations, concerning the hiftory and progrefs of medical fcience, has lately publifhed a fmall pamphlet on Galvanifm and its medical application; in which, after giving a brief but judicious furvey of Galvanifm, he concludes with relating the phenomena which are produced by the effect of Volta's column or pile on the human body, and pointing out the difeafes in which it may be fuccefsfully employed. We fhall only communicate to our Readers the laft part of the above publication, as we have already commenced a more ample hiftorical account of the Galvanic Difcovery than we find in Dr. A's prefent work.

"When a pile is properly conftructed, fo as to fhow itfelf perfectly efficacious, a moft fenfible ftroke will be felt, on touching with wet hands, or by means of filver fpoons, the upper and lower extremities of the pile; the fenfation, however, does not extend itfelf, nor penetrate into the breaft fo much, as from an electrical ftroke; but when the chain is continued to be fhut, the fenfation becomes more permanent and difagreeable, which likewife takes place on putting each chain into a glafs of water and holding each hand in one of the glaffes. Thefe fenfations, however, prove more lively when the pile is frefh conftructed, but decreafe the longer it ftands, particularly when the ends of the pile have been combined with each other by the contact of the two chains. It is however fingular, that the force of the pile may be renewed and reftored by fhaking the chains. The pungent pain perceived on touching both ends of the pile with wet fingers increafes, if he, who touches them, is infulated. When feveral perfons take hold of one another by the hands, which muft be wet, and when thofe at the two ends touch the two extremities of the pile, all of them receive a flight fenfation, which is not fo ftrong as in a fingle perfon, though it becomes ftronger if they are infulated. The commotions become evidently more lively on moiftening the fingers with which the extremities of the pile are touched with a folution of common falt, but they are the moft forcible if a part of the touching fingers is deprived of its epidermis. Galvanifm, applied by means of Volta's pile, fhows a moft fenfible effect on the organs of fenfe, and particularly on the eyes; for on applying the two wires of the

### Galvanic Apparatus with its Medical Application.

Vide page 242. Vol. 7.

### Mr. Reece's Odontagra.

Vide page 221.

the zinc and filver fide on the fkin beneath the eye-lids, which has been previoufly moiftened with falt water, a burning pain will arife, and the light be put into a vibrating motion alternately in both eyes, which continues as long as the wires remain at the above place. The fame phenomenon appears, when we touch with one hand a wire that is placed in a glafs of water, whilft we apply the wet eye-lid to the metallic plate of the upper extremity of the pile. The vibration of light, however, is particularly ftrong on applying one wire on a fpot above the eye-brow that has been previoufly deprived of its epidermis, and on putting the other wire into the nofe or mouth, as the flafhes of lightning and the ftrokes, which then extend themfelves through the whole head, are frequently fo violent and ftupefying, as to caufe lipothymies, when the pile confifts of twenty or thirty ftrata. The other organs of fenfe are likewife evidently affected from Galvanifm by means of Volta's pile. On bringing, for inftance, the wire, *a* Fig. 1, into the ear that has been previoufly moiftened, whilft the other wire, *b*, fig. 1, is held by the hand in a glafs of water, a ftroke and a ftrong found will be perceived in the ear, which becomes more benumbed if the wire *b* is taken out of the water and applied to the other ear, fo as to bring both into the connexion of the chains. On twifting wire about the ears, moiftened with falt water, fo that the ends of the wire can be immerfed into glaffes of water, in which the chains faftened at the extremities of the pile are placed, a giddinefs is occafioned, and moft beautiful flafhes of lightning will be feen. The fenfations of found, however, and tingling in the ear, become particularly ftrong when we put a conductor applied to the Euftachian tube in combination with a chain. On dipping a finger into one of the above glaffes and into the other a zinc bar, and touching this with the tongue, infupportable pains will be felt, a flafh of lightning be feen, and a fingular fourifh tafte remain fome time after. Thefe phenomena, however, are found to differ remarkably by the manner in which the chains are touched, whether by the filver fide or by the zinc fide of the pile, or whether they are connected or feparated; becaufe, in cafe of either fhutting or disuniting the chains, the feelings arifing from the filver fide and that from the zinc fide are always oppofite to one another; and what is moft curious, the fenfation which is occafioned on fhutting the chains at the filver fide is exactly the fame at the zinc fide, when the chains are disjoined, while the feeling at the filver fide changes into that which was perceived on the zinc fide when the chains were fhut, and vice verfa. Thus, for inftance, in the ftrokes perceived in the fingers, the finger at the zinc wire *b*, of

Volta's pile, feels a fenfation as if a ftring was tied round it, whereas a difagreeable pungent feeling paffes through the finger at the filver wire from the point of contact in all directions; but on disjoining the chain this fenfation changes, fo that what the finger at the filver wire felt, is then felt by the finger of the zinc wire, and vice verfa. When the circle or chain is formed, by means of the tongue, by bringing it into contact with the filver wire *b*, fig. 1, of the battery, whilft one end is applied to the zinc wire a very ftrong fhock will be perceived in the tongue, from which an impreffion remains at the fpot where the fhock took place. On forming the chain with the tongue, applied at the filver fide, a very difagreeable fhock is produced, which leaves behind it a feeling as if a hole was ftruck into the tongue. When the tongue is brought in contact with the zinc fide, and the circle formed by another part of the body, a fenfation of heat will be felt, together with the fhock and the fourifh tafte, which, however, on disjoining the chains, is changed into the oppofite fenfation of cold, and vice verfa, when the tongue is in contact with the filver fide. The fenfations produced in the nofe differ likewife according to the manner of applying the wire; when we apply the filver wire, an inclination to fneeze will arife, which is not the cafe with the zinc wire. On bringing the ear in contact with the zinc fide of the battery, a clear found is perceived, which becomes ftronger when the ear is brought in contact with the filver fide. The phenomena produced in the eyes differ alfo according to the different manner in which the eye is brought in contact with Volta's pile.

From thefe ftatements, it appears how much the medical ufe of Galvanifm deferves to be recommended, and that, notwithftanding the few practical obfervations which have hitherto been made on that fubject, we are entitled to expect no inconfiderable advantages from fo powerful and penetrating a ftimulus. In employing it, however, for the cure of difeafes we fhould always confider the topical as well as the general ftate of irritability, in order to prevent any bad confequences which may arife by applying too ftrong a degree of this ftimulus. A ftrong Galvanic fhock generally occafions laffitude and a kind of lamenefs, which continues for a whole day, particularly if we have for any long time expofed ourfelves to the action of the battery. Thus, Mr. Rutter, a gentleman to whofe ingenious experiments we are particularly indebted for many interefting explanations with refpect to this fubject, felt a general indifpofition attended with wearinefs and dullnefs in the head, after having expofed himfelf for a whole hour to the action of a ftrong battery, which confifted of 100 ftrata. Inflammations tions of the eyes after continued experiments with light, debilitated infenfibility of the tongue, catarrhs after frequent experiments in the nofe, vertigo and head ach after violent ftrokes through the head, and tooth-ach, which always enfue after any experiment being for fome time continued at any of the above parts of the head, are the common confequences of Galvanifing, which undoubtedly arife from this ftimulus having acted too violently on the found degree of irritability. In difeafes, therefore, where a great irritability prevails, attended with debility, we ought only to employ it in a weak degree; but in paralyfes from indirect debility we may immediately begin with violent commotions. Regard fhould alfo be had to the organ on which we intend to act. The weakeft degree of Galvanifm is produced by means of two fmall metallic plates, which are placed near each other on any part of the body, that has been previoufly deprived of its epidermis.——The difeafes in which the application of Galvanifm may be attended with fuccefs, are the following:

1. *Afphyxia and apparent death.* Mr. Creve has already recommended, as the fureft method of examining the real or apparent death of a perfon, to apply one branch of an arch, confifting of two metals, to a brachial nerve, previoufly laid bare, and the other branch on a neighbouring mufcle, in order to excite powerfully by this fimple Galvanic chain the debilitated irritability. The coating of the nerve, however, being not neceffary, as a flight wound of the fkin is fufficient for admitting the action of Galvanifm, fmall incifions made with a fcarificator will fully anfwer the above purpofe. To thefe incifions the Galvanic power is to be led by means of the two conductors *c c.* fig. 1, which muft be applied at a fmall diftance from one another. On perceiving convulfions, we may conclude that there is ftill incitability left in the body; but when no commotions are produced by the ftrongeft Volta's pile, a total want of vital power muft be fuppofed, provided the battery is in fuch ftate as to fhew itfelf perfectly efficacious. The fame method may be obferved in afphyxia; we ought, however, always to begin with a few ftrata, and to increafe them by degrees with great precaution, for fear of extinguifhing the fmall quantity of vital power that may be ftill exiftent. For this purpofe, Volta's battery feems by far to be better calculated than any other manner of applying electricity, becaufe the neceffary degree of ftimulus can be eafier adapted to the degree of incitation by Volta's pile; befides, its conftituent parts are more portable than an electrical machine.

2. *Paralyfes.* Under this great clafs of nervous difeafes, we can only comprehend fuch as do not arife from organic defects, fects, as, a preſſure of the nerve, a luxation of the vertebrae lumbares, or a kyphoſis, but which originate in an internal indiſpoſition of the nerves; whence, for inſtance, the common hemiplegies from indirect aſthenia and any topical debility or inactivity of the nervous ſyſtem. In order to apply the Galvaniſm in a paralyſis of the extremities, two ſmall ſpots above the place where the nerve runs, viz. at the thigh above the iſchiatic nerve, and at the arm above the nervus cutaneus externus, are deprived of their epidermis; and when they are ſufficiently moiſtened, the Galvaniſm of Volta's battery is conducted to them by means of the two chains *a b*, and the conductor *c c*, which muſt be taken by the glaſs tube and moved on the excoriated places. When the paralyſis is attended with a high degree of inſenſibility, or a total loſs of motion as well as ſenſation, the Galvaniſm ſhould be applied in a ſtrong degree, in order to excite the nervous power, and to accelerate the proceſs of life by this new and penetrating ſtimulus; which being done, the number of ſtrata muſt be afterwards diminiſhed, ſo that by degrees a leſs degree of this ſtimulus acts with the ſame force as a greater one did before. By obſerving this rule, I ſucceeded ſome time ago in reſtoring ſenſation perfectly, and motion for the moſt part, with cauſing at firſt ſtrong commotions by a battery of ſixty ſtrata in a hemiplegia from indirect debility, attended with total inſenſibility, in a patient ſixty-ſix years of age; but as the limb was vehemently convulſed, and became ſo painful to the patient that ſhe refuſed a continuation of theſe experiments, the cure could not be performed merely by Galvaniſm, but recourſe was had to the uſe of other excitant remedies.

3. *Nervous diſeaſes from direct aſthenia*, in which the irritability is ſo much accumulated, that all ſtimuli produce too violent and preternatural ſenſations; of this kind are, ſpaſms, convulſions, chorea St. Viti, but particularly triſmus and tetanus. The firſt application of a powerful ſtimulus ought naturally to be weak in theſe diſeaſes, and which is to be done by means of two different metallic plates, which being faſtened to a leather ſtrap, are applied at two places, and brought into contact with each other by means of the conducting arch, fig. 4. Theſe places, however, muſt have been previouſly excoriated by bliſters, or wounded by the ſcarificator. When no effect enſues on the application of theſe ſingle plates, we ought to combine them with one another, and thus increaſe by degrees the number of ſtrata and the force of Galvaniſm. By immediately beginning with ſtrong Galvanic ſhocks in theſe directly aſthenic diſeaſes, we might probably do more harm than good; and I ſaw violent pains and convulſions ariſe from from the application of a ftrong Galvanic battery with forty or fifty ftrata, in a patient affected with a continual motion and a great fenfibility of the lower extremities; whereas the ufe of Galvanifm, from a few ftrata, feemed perfectly to agree with him. The application of this ftimulus in cafes of trifmus and tetanus has not yet occurred to me; but it may be fuppofed, in all probability, that thofe difeafes arifing from a total torpor in the mufcles, which originates in the nerves, may be removed by a ftimulus that fhews itfelf fo powerful in fimilar affections. I fhould, however, think it advifable, in this cafe, to increafe the force of the battery more fuddenly.

4. *Weaknefs of fight and amaurofis.* Galvanifm muft be employed here with the utmoft precaution, particularly in the firft cafe. One of the chains, Fig. 1, *b*, being placed in a glafs of water, into which the patient is ordered to hold his hand, while the conductor of the chain, *a*, is applied to the eye-lid, which muft be previoufly moiftened. The flafhes of lightning which then appear, become ftronger or weaker according to the number of ftrata of which the battery confifts; but we fhould begin with a few ftrata, and only increafe them by degrees. In a perfect amaurofis, however, we fhould ufe a more violent degree of Galvanifm; to which end, a blifter being applied above the eye-brows of the difeafed eye, near the glabella, the patient is ordered to put a wire, fig. 1, *b*, into the mouth or the nofe at the fide of the difeafed eye, and the conductor of the chain, fig. 1, *a*, is then brought in contact with the place deprived of its epidermis by means of the blifter. For the beginning, ten ftrata are fufficient, which may be afterwards increafed. Patience and perfeverance are required for performing fuch a cure, and I fucceeded in this cafe, though not till after an experiment eight times repeated, in producing fome change in the difeafed eye; for after having increafed the ftrata to thirty-five, at the ninth experiment a flight glance of light was produced, which is flowly augmenting. I have not experienced any quicker effect of Galvanifm in amaurofis, and I am therefore inclined to think the accounts of amaurofis being cured by Galvanifm in a fhort fpace of time exaggerated, though it may prove of quicker and better effect in the beginning of an amaurofis. There is, however, a kind of amaurofis, which originates from a congeftion of blood towards the head, where Galvanifm is by no means indicated, but where topical venæfection, foot-baths, and the application of cold water on the eye are of the beft fervice.

5. *Difficulty of hearing and deafnefs.* As thefe affections arife from different caufes, in which Galvanifm is of no ufe, we can only expect advantage in thofe cafes where a paralyfis of of the acouftic nerve is to be removed, and perhaps alfo where the fecretion of the ear-wax is to be promoted. In total deafnefs from a paralytic ftate of the acouftic nerve, I ufe immediately in the beginning a battery of twenty or thirty ftrata; but in difficulty of hearing, a lefs degree of Galvanifm is to be applied. The beft mode of application in the above affections is the following: A place behind each ear, on the proceffus maftoideus, being deprived of its epidermis, a zinc plate is applied on one fide and a filver plate on the other, by means of the bandage reprefented in Fig. 5. This bandage confifts of a hoop of whalebone, which is faftened by means of a cap round the occiput, and of a ribband tied under the chin; on the two ends of this hoop we can fcrew the plates, fig. 5, *a*, which being brought in combination by means of a filver chain are fuffered to lie for feveral days: A humming found arifes now in both ears, and an acrid ferum iffues from the fore places, particularly from that touched by the zinc plate, which ftrongly calcines, and on account of its violent effect muft be frequently changed with the filver plate. This mode of applying a fimple Galvanic chain is frequently efficient enough, as a powerful ftimulus, in deafnefs from metaftafes of a morbid matter, and in that kind of difficult hearing which arifes from a drynefs of the internal furface of the tympanum, to be diftinguifhed by the patient's hearing better through the mouth than through the auditory canal, and by the drynefs of the membrana pituitofa of the nofe. In that kind of deafnefs, however, which is attended with a want of the ear wax, it is rather advifable to bring the conductors of Volta's battery, fig. 1, *c c*. into the auditory canal, which muft be previoufly moiftened; for this purpofe we can make ufe of the apparatus, fig. 5, *b*. confifting of a brafs cylinder or nut, fig. 5, *b*. over which the conductor is moved into the ear as far as we think proper. The ftrongeft action of Galvanifm, on the organ of hearing, is produced by bringing a curved wire, fig. 6, into the Euftachian tube, which being put in connection with one chain, the patient is ordered to hold the other in his wet hand.

6. *Aphonia and hoarfenefs*. Thefe affections may alfo originate from different organic lefions, viz. abfciffion of the nervus recurrens, &c. in which Galvanifm is of no fervice; but where obftructions in the glands and a paralyfis of the above nerve, or of the mufcles of the tongue, take place, it proves undoubtedly of great effect. To this end a leather ftrap, fig. 3, may be ufed, by means of which the zinc and filver plates are faftened on two fpots, deprived of their epidermis above the mufculus fternocleido maftoideus, near the larynx: the plates are brought into connection by the conducting metallic arch, fig. 4, which which can be widened and ſhut at *a*. The effect of Galvaniſm may be increaſed by applying the two conductors, fig. 1, *c c*. Dr. Grapengieſſer † ſucceeded in this manner in removing, in the ſpace of twenty-four hours, an aphonia that had laſted ten years.

7. *Chronical rheumatisms with stiffness of the limbs and joints.*

8. *Cold swellings and asthenic inflammations.*

9. *Tooth-ach.*

10. *Oedema and dropsy*; in which the activity of the abſorbent veſſels is to be reſtored or increaſed.

On the whole, we may avail ourſelves of Galvaniſm in any aſthenic diſeaſe; but we ſhould always regard the difference between direct and indirect aſthenia, if we intend to do any good by the application of that powerful ſtimulus.

---

### EXPLANATION OF THE PLATE.

Fig. 1. Volta's battery between four glaſs pillars, with the diſcharging chains, *a b*, and the conductors, *c c*, faſtened to them, which conſiſt of wires paſſing through glaſs tubes.

Fig. 2. One of the braſs plates, which are placed, one on and the other under the pillars, and to which the chains, *a b*, fig. 1, are faſtened.

Fig. 3. Two metallic plates, the one of zinc the other of ſilver, which are faſtened by buttons in a leather ſtrap, to be applied in aphonia and hoarſeneſs.

Fig. 4. The conducting arch, by means of which the connexion between the two plates is formed.

Fig. 5. A bandage for the head, for the application of Galvaniſm in caſes of deafneſs, conſiſting of a piece of whalebone to paſs over the back of the head, which is to be faſtened by a cap on the head and a band under the throat; to the two ends of which two plates of different metal can be ſcrewed over the proceſſus maſtoideus, and the apparatus above deſcribed.

Fig. 6. An inſulating wire paſſing through a glaſs tube, the upper end of which is provided with a button, and conſiſts of fine ſteel, in order to bring it conveniently into the Euſtachian tube.

---

### *To the Editors of the Medical and Phyſical Journal.*

GENTLEMEN,

THE *laſt* number of *The London Medical Review and Magazine*, having given a general invitation to its Correſpondents in the Magazine department, to tranſmit their future Commu-

† We purpoſe to lay before the public, in a future number, the experiments of this gentleman with Volta's battery.

 nications

## Figures and Tables

**Fig. 1. Fig. 2. Fig. 3. Fig. 4. Fig.5. Fig. 6. f1:**